# A retrospective study: the prognostic value of anemia, smoking and drinking in esophageal squamous cell carcinoma with primary radiotherapy

**DOI:** 10.1186/1477-7819-11-249

**Published:** 2013-10-01

**Authors:** Fang Zhang, Hui Han, Chuansheng Wang, Jianbo Wang, Guangyu Zhang, Fangli Cao, Yufeng Cheng

**Affiliations:** 1 Department of Radiotherapy, Cancer Centre, Qilu Hospital, Shandong University, 107 Wenhuaxi Rd, Jinan, Shandong 250012, China; 2 Cancer Centre, Liaocheng People’s Hospital, Liaocheng, Shandong 252000, China; 3 Cancer Centre, The First Hospital of Zibo, Zibo, Shandong 255200, China

**Keywords:** Anemia, Survival, Smoking, Drinking, Squamous cell carcinoma, Esophageal neoplasms

## Abstract

**Background:**

Few studies have investigated the relationship between anemia, smoking, drinking and survival in esophageal squamous cell carcinoma (ESCC) with primary radiotherapy. This study had the aim of evaluating the prognostic value of anemia, smoking and drinking in patients receiving primary radiotherapy for ESCC.

**Methods:**

A total of 79 patients who underwent radiotherapy during initial treatment for ESCC were included in this study. The 2-year overall survival (OS) and disease-free survival (DFS) were analyzed between the anemic and non-anemic groups, non-smokers and smokers, and non-drinkers and drinkers using the Kaplan-Meier method and the Cox proportional hazards model.

**Results:**

There were 79 patients (10 male) of median age 63 (range 38 to 84) years. The 2-year OS and DFS were 36% and 25%, respectively, in the non-anemic group, and 17% and 13%, respectively, in the anemic group (*P* = 0.019 for OS; *P* = 0.029 for DFS) using the Kaplan-Meier method. Survival analysis using the Kaplan-Meier method showed that the 2-year OS and DFS had no statistical difference between smoking, drinking and survival. In a univariate analysis, anemia was identified as a significant prognostic factor for 2-year OS (hazard ratio (HR) = 1.897; *P* = 0.024) and 2-year DFS (HR = 1.776; *P* = 0.036), independent of tumor, lymph node, metastasis (TNM) stage. In a multivariate analysis, anemia was identified as a highly significant prognostic factor for 2-year OS (HR = 2.125; *P* = 0.011) and 2-year DFS (HR = 1.898; *P* = 0.025), independent of TNM stage and initial treatment. We found no statistical difference in the 2-year OS and DFS associated with smoking (*P* > 0.2) and drinking (*P* > 0.6) using univariate and multivariate analysis.

**Conclusions:**

Smoking and drinking were not prognostic for 2-year OS or DFS. Anemia before radiotherapy was associated with poor prognosis and an increased risk of relapse, which may serve as a new prognostic characteristic in ESCC treated with primary radiotherapy. Hemoglobin is a routine examination and anemia is therefore simple and quick to determine.

## Background

Esophageal cancer is the eighth most common cancer and the sixth most common cause of mortality in the world [1]. The distribution of esophageal cancer is heterogenous. Developed countries (except for Japan) have low rates, while China has a high rate of esophageal cancer, the fourth most common cause of mortality in China [2,3]. Esophageal squamous cell carcinoma (ESCC) continues to be the major type of esophageal cancer in Asia; in contrast, esophageal adenocarcinoma predominately affects the whites. Tobacco smoking and alcohol are well established causes of ESCC; however, there are few reports that directly evaluate these factors as prognostic factors for esophageal cancer.

Anemia is known to produce tumor hypoxia which confers radio-resistance through the hypoxia-associated reduction in free-radical production and consequent radiotherapy-induced DNA damage [4]. Fein and colleagues reported that the 2-year local control rates in the non-anemic group were significantly better than those in the anemic group (*P* < 0.0018) in T1-T2 squamous cell carcinoma of the glottic larynx [5]. However, few studies have investigated the relation between anemia and survival in patients with ESCC.

The objective of this study was to evaluate the prognostic value of anemia, smoking and drinking in patients receiving primary radiotherapy for ESCC and its relationship with other prognostic factors.

## Methods

### Patients

From the database of the Qilu Hospital of Shandong University, we selected all patients with ESCC who underwent radiotherapy during initial treatment at the Department of Radiation Oncology of the Qilu Hospital from 1 January 2009 to 31 December 2010. All patients who did not undergo operation in this study were staged according to routine practice of our hospital with air contrast barium esophagography, upper gastrointestinal endoscopy with histological biopsies and cervical, chest and abdominal contrast computed tomography. All patients who underwent operation in this study were staged according to the American Joint Committee on Cancer TNM staging system [6]. All patients had intended curative radiation therapy alone or pre- or post-operative radiotherapy or radiochemotherapy according to the practice. Radiotherapy was started on day 1 and delivered at 2 Gy/day for 5 days a week for a total radiation dose of 66 to 72 Gy for those without operation and a total radiation dose of 50 Gy for those with operation. Patients without recorded hemoglobin levels were excluded, as were patients who died during radiotherapy. Patient, tumor, and treatment characteristics were retrieved from the Medical Records Room. All patients signed informed consent to this study, and the protocol was approved by the Ethics Committee of Qilu Hospital of Shandong University.

### Definition of anemia

The definition of anemia used in this study was kept consistent with the definitions used by our laboratory: a hemoglobin level under 12 g/dL for men and under 11 g/dL for women. A hemoglobin level of 9 to 12 g/dL for men and 9 to 11 g/dL for women was defined as mild anemia, and a hemoglobin level of 6 to 9 g/dL for both men and women as moderate anemia. A patient was classified as being anemic if their hemoglobin measured before radiotherapy met these levels. During this study the ‘before-radiotherapy’ was defined as 4 weeks prior to receiving radiotherapy.

### Follow-up

Follow-up data were collected until death or 31 December 2012. All patients had a regular follow-up schedule including a complete history and physical examination every 3 months during the first 2 years, every 6 months during the first 3 to 5 years and every year thereafter. Routine radiological examinations were performed when necessary.

### Statistical analysis

Differences in patient, tumor, and treatment characteristics were assessed using the Mann–Whitney test for continuous variables and the *χ*2 test for categorical variables. The Kaplan-Meier method and log-rank test were used for analysis and comparison of survival curves. For the analysis of 2-year overall survival (OS), events were defined as death from any cause. For the analysis of 2-year disease-free survival (DFS), events were defined as first loco-regional or distant tumor relapse or death from any cause. The Cox proportional hazards model was used to determine the hazard ratio (HR) of variables on 2-year OS and DFS in univariate and multivariate analysis. The results are given as HRs with their 95% confidence interval (CI). *P* values less than 0.05 were considered statistically significant. Data were analyzed using the statistical package SPSS version 17.0 (SPSS Inc., Chicago, IL, USA).

## Results

### The prevalence of anemia, smoking and drinking

There were 23 anemic patients from the total of 79 patients; 20 patients with mild anemia, and 3 with moderate anemia. The median hemoglobin levels were 13.8 g/dL (11.8 to 16.9) in the non-anemic group and 10.6 g/dL (8.3 to 11.9) in the anemic group. The prevalence of anemia in patients undergoing radiotherapy was 29.1%. Of the 79 patients, 33 patients (41.8%) were non-smokers, whereas 46 patients (58.2%) were smokers. Of the 79 patients, 39 patients (49.4%) were non-drinkers, whereas 46 patients (50.6%) were drinkers.

### Clinicopathological features

All patients (69 men and 10 women) were included in this study. The median age of the patients was 63 (range 38 to 84) years at the date diagnosed. The median survival time was 19 (2 to 56) months. Characteristics of the patients are shown in Table 1.

**Table 1:** Patients characteristics between the anaemic and non-anaemic groups

		**Anemic**	**Non-anemic**	***P*value**
Median age (years)		63 (40–76)	63 (38–84)	0.393
Male (n)		18	51	0.145
Female (n)		5	5	
Hemoglobin (g/dL)	Male	10. 5 (8.3-11.9)	13.7 (12.0-16.9)	0.000
	Female	10.7 (9.3-10.8)	14.5 (11.8-15.3)	0.000
Tumor location (n)	Cervical	1	7	0.639
	Upper	3	10	
	Middle	13	26	
	Low	6	13	
Initial treatment (n)	Radiotherapy	3	19	0.134
	Surgery + radiotherapy	8	11	
	Chemoradiotherapy	6	18	
	Surgery + Chemoradiotherapy	6	8	
TNM stage (n)	I	1	6	0.647
	II	6	19	
	III	12	24	
	IV	4	7	
Smoking (n)	No	8	25	0.420
	Yes	15	31	
Drinking (n)	No	10	29	0.502
	Yes	13	27	

TNM, tumor, lymph node, metastasis.

### Correlation of anemia with other prognostic factors

**Figure 1:**
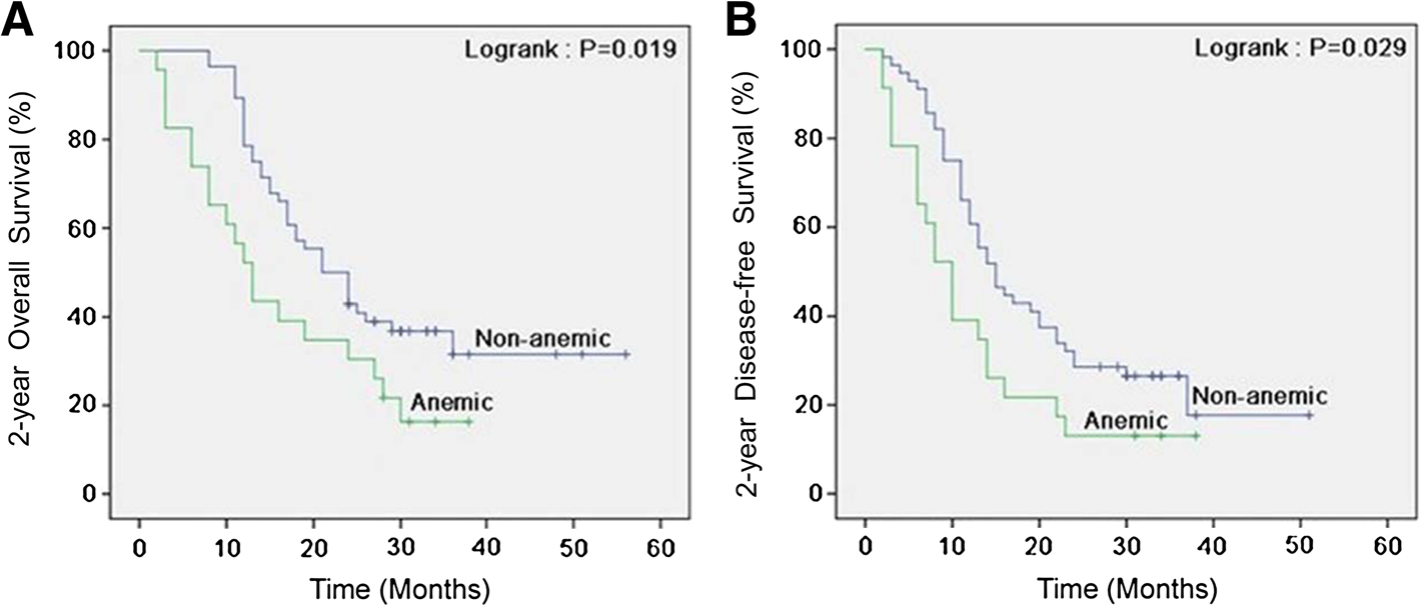
**Kaplan-Meier curves for survival of 79 patients with esophageal squamous cell carcinoma (non-anemic versus anemic).** Both 2-year overall survival **(A)** and disease-free survival **(B)** between two groups remained statistically significant.

Table 1 shows patient characteristics for the non-anemic and the anemic groups. There were no significant differences between the two groups. Follow up was complete. The 2-year OS and DFS were 36% and 25%, respectively, in the non-anemic group, and 17% and 13%, respectively, in the anemic group. Both 2-year OS and DFS in the non-anemic group were significantly better than those in the anemic group (*P* = 0.019 for OS; *P* = 0.029 for DFS) using the Kaplan-Meier method. Survival curves are shown in Figure 1.

In the Cox univariate model, anemia and tumor, lymph node, metastasis (TNM) stage were significantly related to 2-year OS (HR = 1.897, 95% CI 1.086 to 3.312; *P* = 0.024) and DFS (HR = 1.776, 95% CI 1.040 to 3.033; *P* = 0.036), respectively. Anemia before radiotherapy was associated with poor prognosis and an increased risk of relapse (Table 2). In a multivariate analysis, anemia was identified as a highly significant prognostic factor for 2-year OS (HR = 2.125; *P* = 0.011) and 2-year DFS (HR = 1.898; *P* = 0.025), independent of TNM stage and initial treatment (Table 3).

**Table 2:** Cox univariate analysis for 2-year survival in 79 patients

	**Univariate analysis**					
	**2-year overall survival**			**2-year disease-free survival**		
	**HR**	**95% CI**	***P***	**HR**	**95% CI**	***P***
Hemoglobin (non-anemic vs anemic)	1.897	1.086-3.312	0.024	1.776	1.040-3.033	0.036
Smoking (no vs yes)	0.889	0.522-1.514	0.666	0.981	0.593-1.622	0.940
Drinking (no vs yes)	0.941	0.554-1.600	0.824	0.959	0.582-1.579	0.869
TNM stage (I vs II vs III vs IV)	1.652	1.175-2.322	0.004	1.650	1.193-2.281	0.002
Initial treatment (RT vs S + RT vs CRT vs S + CRT)	0.840	0.655-1.077	0.169	0.900	0.718-1.129	0.362
Tumor location (C vs U vs M vs L)	0.937	0.703-1.250	0.660	0.964	0.736-1.263	0.792

C, cervical; CI, confidence interval; CRT, chemoradiotherapy; HR, hazard ratio; L, low; M, middle; RT, radiotherapy; S, surgery; TNM, tumor, lymph node, metastasis; U, upper; vs, versus.

**Table 3:** Cox multivariate analysis for 2-year survival in 79 patients

	**Multivariate analysis**					
	**2-year overall survival**			**2-year disease-free survival**		
	**HR**	**95% CI**	***P***	**HR**	**95% CI**	***P***
Hemoglobin (non-anemic vs anemic)	2.125	1.190-3.796	0.011	1.898	1.085-3.320	0.025
Smoking (no vs yes)	0.623	0.286-1.359	0.235	0.746	0.370-1.503	0.412
Drinking (no vs yes)	1.208	0.559-2.611	0.631	1.170	0.581-2.354	0.660
TNM stage (I vs II vs III vs IV)	1.722	1.231-2.409	0.001	1.653	1.205-2.267	0.002
Initial treatment(RT vs S + RT vs CRT vs S + CRT)	0.732	0.561-0.956	0.022	0.812	0.640-1.032	0.088

CI, confidence interval; CRT, chemoradiotherapy; HR, hazard ratio; RT, radiotherapy; S, surgery; TNM, tumor, lymph node, metastasis; vs, versus.

### Correlation of smoking and drinking with other prognostic factors

Follow-up was complete. Survival analysis using the Kaplan-Meier method showed that there were no significant differences in the 2-year OS and DFS between non-smokers and smokers (*P* = 0.658 for OS; *P* = 0.939 for DFS) and non-drinkers and drinkers (*P* = 0.819 for OS; *P* = 0.866 for DFS). Using univariate and multivariate analysis, there were no statistical differences on survival between the non-smokers and the smokers, and between the non-drinkers and drinkers (Tables 2 and 3).

## Discussion

In this study, the prevalence of anemia in patients with ESCC undergoing primary radiotherapy was 29.1%. Similarly, Walter and colleagues [7] reported that the prevalence of anemia in rectal carcinoma with neoadjuvant radiotherapy was 35%.

Recently, much research has shown that anemia was associated with poor prognosis and an increased risk of relapse. Dietl and colleagues [8] reported that anemia was an independent negative prognostic factor for local recurrence-free survival and overall survival in patients with head and neck cancer treated with surgery and postoperative radiotherapy. Furthermore, moderate anemia was also confirmed as an independent prognostic factor in squamous cell carcinoma of the head and neck treated with radiotherapy alone [9]. Grigiene and colleagues [10] found that the hemoglobin level before treatment was an independent prognostic factor for OS, DFS and local relapse-free survival for patients with uterine cervical carcinoma treated with irradiation using univariate and multivariate analysis. In addition, MacRae and colleagues [11] revealed that a decline in hemoglobin during chemoradiation for stage III non-small cell lung cancer had a statistically significant correlation with OS.

The present investigation shows that anemia is a new independent prognostic factor for esophageal carcinoma. We found that the 2-year OS and DFS were 36% and 25%, respectively, in the non-anemic group, and 17% and 13%, respectively, in the anemic group (*P* = 0.019 for OS; *P* = 0.029 for DFS) using the Kaplan-Meier method. Both 2-year OS and DFS in the non-anemic group were significantly better than those in the anemic group. In a multivariate analysis, anemia was identified as a highly significant prognostic factor for 2-year OS and 2-year DFS, independent of TNM stage and initial treatment. Zenda and colleagues [12] concluded that the pre-chemoradiotherapy hemoglobin level may be an important determinant of outcome in patients with T4/M1 LYM squamous cell carcinoma of the esophagus. This result was consistent with previous studies regarding anemia. Zhao and colleagues [13] revealed that anemia resulted in a statistically significant reduction in OS and DFS for patients with locally advanced esophageal carcinoma undergoing irradiation. In addition, Hofheinz and colleagues [14] reported that a significant decrease in hemoglobin levels during primary chemoradiotherapy was identified as a prognostic factor for ESCC.

Smoking has long been known to be a major risk factor for ESCC, but little is known about the possible influence of the risk factor on survival. In our current study, there were no statistically significant associations between smoking and OS or DFS in ESCC treated with primary radiotherapy using the Kaplan-Meier method (*P* > 0.6). Using univariate and multivariate analysis, our data fail to demonstrate a link with smoking and 2-year OS or DFS for patients with ESCC treated with primary radiotherapy (*P* > 0.2). There have been some similar studies published. In a series of 135 patients, Kandaz and colleagues [15] found no statistical difference on survival based on smoking in 135 patients with locally advanced cancer of the esophagus treated with radiotherapy and/or chemoradiotherapy. Michalek and colleagues [16] analyzed the outcome of 302 patients with bladder cancer and showed that smoking status was not associated with either recurrence-free survival or the number of tumor recurrences. These findings suggest that cigarette smoking is not an important prognostic factor for patients with bladder cancer. The prognostic significance of smoking was analyzed in 242 incident cases of breast cancer from Norwegian women. Again, there was no statistical difference on survival [17].

Drinking has been identified as a major risk factor for esophageal cancer, but the effect on survival is unknown. A study in esophageal carcinoma and cardia adenocarcinoma suggested that there were no statistically significant associations between alcohol consumption and prognosis [18]. Samadi and colleagues [19] reported that drinking did not show any significant effects on the survival rate of patients with gastric and esophageal cancers using the univariate and multivariate analysis. Similarly, our data fail to demonstrate a link between drinking and OS or DFS in patients with ESCC treated with primary radiotherapy.

In our study, we did not show that smoking and drinking were associated with survival in patients with ESCC treated with primary radiotherapy. Trivers and colleagues [20] found that cigarette smoking and alcohol intake did not predict survival of patients with esophageal or gastric cancers. Furthermore, Tsai and colleagues [21] analyzed 797 young patients with esophageal cancer that received esophagectomy and found that there was no statistical difference between smoking, drinking and survival.

Many prognostic factors have been found for esophageal cancer, such as TNM stage, tumor location, and depth of tumor invasion. In this study, we found that TNM stage was significantly related to 2-year OS and DFS using univariate analysis. This result was consistent with a previous study of 797 patients with esophageal cancer who received esophagectomy [21]. For 2-year OS, TNM stage and initial treatment were independent prognostic factors, while, for 2-year DFS, TNM stage was an independent prognostic factor using multivariate analysis. Our results were similar to those of previous studies [13,22,23].

## Conclusion

In summary, we found that smoking and drinking were not prognostic factors for 2-year OS or DFS. Our findings indicate that anemia before radiotherapy is a new prognostic factor for ESCC with primary radiotherapy, independent of other prognostic factors. In addition, hemoglobin is a routine examination, and so anemia can be easily assessed. Further research with larger sample sizes are needed to validate these findings.

## Consent

Written informed consent was obtained from the patients for publication of this research articles and any accompanying images.

## Abbreviations

CI: Confidence interval; DFS: Disease-free survival; ESCC: Esophageal squamous cell carcinoma; HR: Hazard ratio; OS: Overall survival; TNF: Tumor, lymph node, metastasis.

## Competing interests

The authors declare that they have no competing interests.

## Authors’ contributions

ZF conceived the study, participated in its design, performed the statistical analysis and drafted the manuscript. HH, WJ and WC participated in the study design, literature search and coordination. ZG and CF participated in the analysis of experimental results. CY participated in the design of the study and coordination, and helped to draft the manuscript. All authors read and approved the final manuscript.
